# Distributed *H*_−_/*H*_∞_ Attack Detection for Intelligent Connected Vehicle Platoons: A Coupling-Aware Observer Approach

**DOI:** 10.3390/s26216820

**Published:** 2026-10-28

**Authors:** Li Niu, Shenghui Guo

**Affiliations:** 1 School of Computer Engineering, Suzhou Polytechnic University, Suzhou 215104, China; nl@jsspu.edu.cn; 2 School of Electronic and Information Engineering, Suzhou University of Science and Technology, Suzhou 215009, China

**Keywords:** intelligent connected vehicles, platoon, attack detection, *H*_−_/*H*_∞_ index, distributed observer, linear matrix inequality, cyber-physical security

## Abstract

Cooperative driving stacks for intelligent connected vehicles (ICVs) depend on a continuous exchange of position and velocity data over vehicle-to-vehicle links. That dependence is also their weakness: an adversary who tampers with exchanged measurements can push a platoon toward unsafe spacing before any physical symptom appears. This paper develops a distributed attack-detection scheme for ICV platoons that is sensitive to such data manipulations yet remains robust to road disturbances. Each vehicle runs a local observer whose correction term mixes its own sensor residual with the state estimates broadcast by its immediate neighbours, so the wind-resistance coupling between vehicles is exploited rather than ignored. Using a modal decomposition of the platoon dynamics over the communication graph, sufficient conditions for an *H*_∞_ disturbance-attenuation level and an *H*_−_ attack-sensitivity level are derived as a common set of linear matrix inequalities; a bisection algorithm then computes a single observer gain meeting both. Detection rests on a windowed residual-energy test with per-vehicle thresholds calibrated as empirical quantiles over an ensemble of attack-free runs. On a five-vehicle platoon, the detector identifies transient FDI and replay attacks with detection rates of 100% and 100% at a validated false-alarm rate of 0.46%, reaches a mean detection rate of 97.7% over the transient-attack windows of a 300-run Monte Carlo study, and exposes a denial-of-service attack on a single link through the open-loop prediction mismatch it induces (48.8% of the attacked window); stealthy zero-dynamics attacks, which by construction excite the least observable directions of the plant, are flagged on 0.0% of attacked samples in the documented low-energy configuration, while an amplitude–rate–location sweep shows detection climbing to 98% once the attack energy grows, confirming attack energy rather than placement as the operative limit. Relative to an *H*_∞_-only observer, it trades empirical detection aggressiveness for a structural guarantee: a certified worst-case attack-sensitivity floor that the baseline does not provide and that survives coupling levels at which uncertified designs lose feasibility. A closing experiment embeds the detector in a cooperative adaptive cruise control loop: mean peak spacing error during a sensor attack averages 7.93 m, against 10.79 m without detection, while forced false-alarm rates of 5–15% latch the conservative control schedule and degrade spacing beyond the no-detection case; a calibrated low false-alarm rate is a safety property, not a convenience.

## 1. Introduction

Vehicle platooning is one of the few intelligent transportation applications in which the benefits are visible at the level of the traffic stream rather than only the individual driver. When vehicles follow one another at short but controlled gaps, using vehicle-to-vehicle (V2V) communication to supplement onboard ranging sensors, road capacity rises and the aerodynamic drag of each vehicle falls; both effects have been quantified since the early field operational tests of cooperative adaptive cruise control (CACC) [1], and the supporting wireless architectures were surveyed in [2]. On the horizon, large language models (LLMs), agentic AI, and embodied AI are reshaping vehicular communications, as charted comprehensively in the recent survey by Wu et al. [3]. Platoon control under communication impairments has since become an active line of work, including event-triggered schemes that tolerate intermittent denial-of-service (DoS) attacks [4]; the broader attack surface of connected and automated vehicles is mapped in the survey by Ju et al. [5].

The same radio links that enable coordination can be abused. Petit and Shladover catalogued realistic attack entry points in automated vehicles as early as 2015 [6], and subsequent work has examined data manipulation in internet-of-vehicles storage and key management [7], resilient control of power networks subject to DoS and false data injection (FDI) attacks [8], and secure fusion estimation under FDI attacks [9]. For platoons, the stakes are higher than for a single vehicle, because one corrupted measurement is propagated downstream by every following vehicle that trusts it. An undetected bias on a velocity sensor can therefore grow into a spacing violation several vehicles away within seconds.

A number of detection and resilient-control strategies have been proposed for connected vehicles. Observer-based sliding-mode control that accommodates DoS attacks was developed in [10], distributed adaptive platooning over vehicular ad hoc networks in [11], and resilient control with full-state constraints under actuator faults in [12]; a resource-aware control scheme that reacts to FDI attacks appears in [13]. Detection-oriented methods include the sliding-mode observer of Biron et al. for DoS attacks on connected vehicle systems [14], the resilient estimation and detection framework for compromised measurements by He et al. [15], output-only fault classification [16], and distributed fault-tolerant tracking control [17]. What these works share is a focus on either robustness to disturbances or sensitivity to attacks. The two requirements pull in opposite directions, and few platoon-specific designs treat them simultaneously.

The *H*_−_/*H*_∞_ framework was created precisely for this tension. The mixed *H*_−_/*H*_∞_ observer design of Wang et al. [18] showed that a single filter can guarantee a prescribed disturbance-attenuation level while keeping a minimum sensitivity to faults, and the idea was later extended to switched systems with interval time-varying delays [19], interconnected systems with sensor faults [20], and cyber-physical power systems under attack [21]. Within the vehicle literature, related ideas have appeared in set-membership estimation for lane keeping [22], finite-time attack detection for platoons [23], and trust-level monitoring inside a zero-trust architecture [24]. None of these, however, addresses the robustness–sensitivity trade-off for platoon attack detection with a distributed, coupling-aware observer. Most relevant to the present work are the distributed Kalman-filter/GLR detector for sensor deception attacks in platoons of Ju et al. [25], the finite-time *H*_∞_ filter for platoon attack detection of Wu et al. [26], and the finite-time distributed detector of [23]; these works target stochastic settings with known noise statistics or certify finite-time convergence of the estimation error, whereas the present design certifies, in a deterministic worst-case sense, simultaneous disturbance attenuation and attack sensitivity with a single common gain, and makes the robustness–sensitivity trade-off explicit.

This paper addresses that trade-off. The platoon model retains the inter-vehicle coupling induced by aerodynamic drag, and each vehicle estimates its own state with a Luenberger-type observer that consumes the broadcast estimates of its neighbours. The graph structure invites a modal decomposition: the platoon error dynamics split into N modal systems, one per Laplacian eigenvalue, and the *H*_∞_ and *H*_−_ requirements reduce to the same inequalities repeated at each mode. The main results are three theorems. Theorem 1 gives a linear matrix inequality (LMI) condition for a prescribed *H*_∞_ level, Theorem 2 gives an LMI condition for a prescribed *H*_−_ level, and Theorem 3 states that one observer gain satisfies both whenever a common Lyapunov matrix satisfies both. A practical point is stressed in the statements: without a bound on the observer gain the *H*_∞_ optimum is trivially approached by an infinitely fast observer, so the conditions include an explicit gain bound that reflects implementation limits. A bisection algorithm computes the gain.

The detector is validated on a five-vehicle platoon under four attack classes: transient FDI, zero-dynamics attacks of the kind introduced by Mo et al. [27] and studied for covert appropriation by Smith [28], DoS attacks on a single V2V link [29], and replay attacks together with the watermarking countermeasures discussed in [30,31]. Against an *H*_∞_-only observer tuned for robustness alone, the mixed design certifies a prescribed attack-sensitivity level jointly with the disturbance-attenuation level, a certificate the baseline does not provide, while Monte Carlo runs quantify the variability of both. A closing experiment embeds the detector in a CACC loop to show that trustworthy alarms are what make attack-triggered controller switching pay off in practice.

The contributions can be summarised as follows:

1. A distributed observer for ICV platoons is proposed whose correction term explicitly uses neighbour estimates, and a distributed-observability lemma certifies that such observers can work with local measurements only. The value of the coupling term is assessed by ablation in Section 4.6: it already decides denial-of-service detection at the nominal coupling (the decoupled observer’s recalibrated thresholds must absorb the unmodelled drag term, and its detection collapses), and it becomes decisive at stronger coupling, where the coupling-aware observer keeps a 100% transient-attack detection rate while the decoupled observer falls to 81% and then 28% and loses its certificate.

2. Modal *H*_∞_ and *H*_−_ conditions are derived with a common Lyapunov matrix, a gain bound that removes the trivial high-gain optimum, and a bisection algorithm that returns a single gain satisfying both indices; the resulting trade-off curve between the two indices is displayed explicitly.

3. The detector is benchmarked against four attack classes with reproducible parameters, 300-run Monte Carlo evaluation, receiver operating characteristic analysis, ablations, and a closed-loop CACC study with false-alarm control groups. A zero-dynamics parameter sweep, a packet-loss robustness study, and a freeze-on-alarm adaptive threshold complete the evaluation.

Section 2 fixes the notation and states the platoon model, the attack model, and the detection problem. Section 3 contains the observer design, the three theorems, the algorithm, and the residual-based decision logic. Section 4 reports the simulation study, Section 5 discusses limitations, and Section 6 concludes.

## 2. Preliminaries and Problem Statement

### 2.1. Notation

In this paper, ℝ^n^ and ℝ^n×m^ denote the n-dimensional Euclidean space and the set of real n × m matrices. *I*_n_ is the n × n identity matrix, 1_N_ is the N-dimensional all-ones vector, and ⊗ is the Kronecker product. For a square matrix M, He{M}: = M + M^T^; M ≻ 0 (M ≺ 0) means positive (negative) definite. ∥⋅∥ is the Euclidean norm. The state of vehicle i is

$$x_{i}={\left[\begin{array}{ccc} p_{i} & v_{i} & a_{i} \end{array}\right]}^{T}$$

with position p_i_, velocity v_i_, and acceleration a_i_. A communication graph G(Γ) with adjacency entries Γ_ij_ ≥ 0 is associated with the platoon; its Laplacian L_Γ_ satisfies [L_Γ_]_ij_ = −Γ_ij_ for i ≠ j and $[L_{\Gamma }{]}_{\mathrm{ii}}=\sum_{j\neq i}\Gamma_{\mathrm{ij}}$. For a path graph of N vehicles, the Laplacian eigenvalues are known in closed form:

$$\lambda_{k}=2-2\cos\left(\frac{k\pi }{N}\right),\;k=0,1,\ldots ,N-1$$

### 2.2. Platoon Dynamics

Consider a platoon of N ICVs travelling in a single lane, indexed 1 (front) to N (rear). Vehicle i measures its own position and velocity, receives the broadcast state estimates of its neighbours, and computes its control input locally. The longitudinal dynamics of vehicle i are modelled as

(1) $${\dot{x}}_{i}(t)=Ax_{i}(t)+Bu_{i}(t)+D_{d}d_{i}(t)+\sum_{j\neq i}\Gamma_{\mathrm{ij}}H\left(x_{j}(t)-x_{i}(t)\right)$$

(2) $$\begin{array}{r} A=\left[\begin{array}{ccc} 0 & 1 & 0\\ 0 & 0 & 1\\ 0 & 0 & -\sigma \end{array}\right],\;B=\left[\begin{array}{c} 0\\ 0\\ \sigma \end{array}\right],\;D_{d}=\left[\begin{array}{c} 0\\ \vartheta_{1}\\ \vartheta_{2} \end{array}\right],\;H=\left[\begin{array}{ccc} 0 & 0 & 0\\ l & 0 & 0\\ 0 & 0 & 0 \end{array}\right] \end{array}$$

where u_i_ is the control input, d_i_ is an unknown external disturbance, and the matrices carry a simple physical reading: the third row of (1) is a first-order engine-lag model ${\dot{a}}_{i}=-\sigma a_{i}+\sigma u_{i}$ with time constant 1/σ, and the matrix H couples vehicle j into the velocity equation of vehicle i through the relative-position term 𝓁(p_j_ − p_i_), a lumped representation of the drag reduction (or increase) felt behind another vehicle. The disturbance enters the velocity and acceleration channels with weights ϑ_1_ and ϑ_2_. Throughout, G(Γ) is assumed undirected and connected, with Γ_ij_ = Γ_ji_. With this parameterisation the engine pole sits at −σ, so the time constant is 1/σ (two seconds for σ = 0.5, as in Section 4.1) and the DC gain from u_i_ to a_i_ is one.

**Figure 1:**
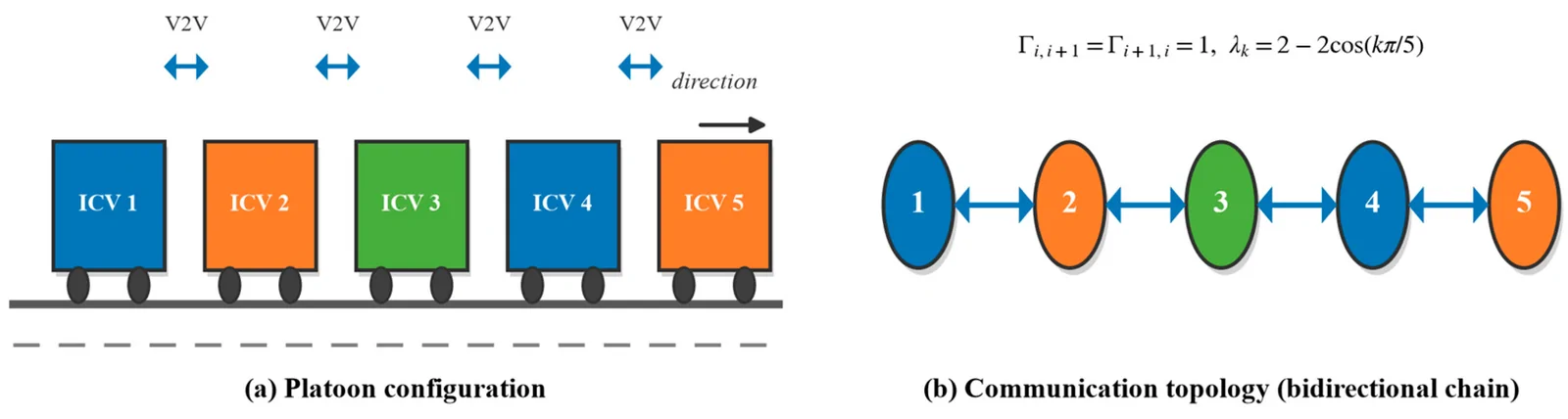
(**a**) ICV platoon with V2V links; (**b**) communication topology: a bidirectional chain with unit adjacency weights, whose Laplacian eigenvalues λ_k_ = 2 − 2cos(kπ/N) drive the modal design of Section 3.

The model is deliberately low-dimensional; higher-fidelity powertrain maps can be absorbed into the uncertainty channels, and the *H*_∞_ condition of Section 3 then covers the neglected dynamics to the extent that they act through D_d_. Figure 1 illustrates the platoon configuration and the communication topology used throughout the paper.

### 2.3. Attack Model

The measurement that vehicle i uses for estimation and control is

(3) $$\begin{array}{r} y_{i}(t)=Cx_{i}(t)+Ef_{s,i}(t),\;C=\left[\begin{array}{ccc} 1 & 0 & 0\\ 0 & 1 & 0 \end{array}\right],\;E=\left[\begin{array}{c} 0\\ 1 \end{array}\right] \end{array}$$

where f_s,i_ is a sensor attack injected into the velocity channel. Four attack classes are considered, all energy-bounded in the sense of Assumption 3 below.

Transient FDI attacks: Piecewise continuous, bounded signals of sinusoidal or step shape on a subset of vehicles over prescribed windows. They represent the most common test case in the platoon literature [23] and are easy to generate, which makes them useful as a baseline.

Zero-dynamics attacks: Following the constructions in [27,28], the attacker drives the zero dynamics of the triple (A,B_a_,C), with B_a_ = (0,1,1)^T^ driven by a small internal state so that the attack signal itself decays quickly while the plant state is displaced along an unobservable direction; for this platoon, the zero-dynamics subspace is one-dimensional (unstable in the classical constructions of [27,28]; here, the zero is stable, sitting at −(σ + 1)). The attack signal is injected through the measurement map E as in (3); the feedthrough term E passes the attack directly to the residual, so the construction is a fast-decaying approximation of an ideal zero-dynamics attack rather than an exactly invisible one. The geometric tools used to compute it are standard [32], Ch. 5. The residual signature of such attacks is orders of magnitude smaller than that of a transient attack of comparable effort, which is exactly why they are used to stress-test detectors.

DoS attacks on a V2V link: The adversary blocks the transmission from vehicle j to vehicle i over an interval; vehicle i then predicts the missing neighbour state in open loop, as in the prediction-based treatments of DoS for platoons [29]. The detector sees the prediction mismatch rather than the attack itself, a distinction that matters when interpreting alarms.

Replay attacks: The attacker feeds back previously recorded measurements of vehicle i [30]; because the platoon state has moved on, the replayed data is inconsistent with the neighbours’ estimates. Sequential detectors tailored to replays exist [31] and could be combined with the present scheme; here, replay is used as a black-box test of the residual-based decision.

### 2.4. Problem Formulation

**Assumption** **1.** *The graph* G(Γ) *is undirected and connected*.

**Assumption** **2.** *The disturbance is energy bounded: *$\int_{0}^{\infty }∥\;d_{i}(t){∥}^{2}\mathrm{dt}<\infty$* for all* i.

**Assumption** **3.** *The attack signal is energy bounded: *$\int_{0}^{\infty }∥\;f_{s,i}(t){∥}^{2}\mathrm{dt}<\infty$* for all* i.

The following lemma shows that local observers have enough information to work with. It is a platoon-grade version of the standard PBH argument; the Kronecker-product form of the PBH test used in the proof is classical [33].

**Lemma** **1 (distributed observability).** *Stack*

$$x={\left[\begin{array}{cccc} x_{1}^{T} & x_{2}^{T} & \ldots & x_{N}^{T} \end{array}\right]}^{T}$$

*and define*

*A_g_* = *I_N_* ⊗ *A* − *L_Γ_* ⊗ *H*, *C_g_* = *I_N_* ⊗ *C*.

*The pair (C_g_,A_g_) is observable if and only if each pair (C,A) is observable; connectivity of G(Γ) is not required*.

**Proof of Lemma** **1. (If.)** Consider first the connected case; if *G*(Γ) is disconnected, the same block argument applies to each connected component whose zero-eigenvalue block is the local pair itself (see Remark 1). Each (C,A) is observable, so by the PBH test $\mathrm{rank}\left(\left[\begin{array}{c} \mathrm{sI}-A\\ C \end{array}\right]\right)=3$ for every s ∈ ℂ. For the global pair, note that

sI − A_g_ = (U ⊗ I_3_)(I_N_ ⊗ (sI − A) + Λ ⊗ H)(U^T^ ⊗ I_3_)

with U from the spectral decomposition L_Γ_ = UΛU^T^. Hence, sI − A_g_ is similar to a block-diagonal matrix with blocks sI − A + λ_k_H. Each block satisfies $\mathrm{rank}\left(\left[\begin{array}{c} \mathrm{sI}-A+\lambda_{k}H\\ C \end{array}\right]\right)=3$ for all s, because (C,A − λ_k_H) inherits observability from (C,A): the modal state matrix differs from A only by the single entry −λ_k_𝓁 in the (2,1) position, and the observability determinant is unchanged. Therefore, $\mathrm{rank}\left(\left[\begin{array}{c} \mathrm{sI}-A_{g}\\ C_{g} \end{array}\right]\right)=3N$ for all s, and (C_g_,A_g_) is observable.

(Only if.) Suppose (C,A) is not observable. Then, there exist s and v ≠ 0 with Av = sv and Cv = 0. Since L_Γ_1_N_ = 0, the vector 1_N_ ⊗ v satisfies

A_g_(1_N_ ⊗ v) = 1_N_ ⊗ Av = s(1_N_ ⊗ v), C_g_(1_N_ ⊗ v) = 0,

so s is an unobservable mode of the global pair, a contradiction. If G(Γ) is disconnected, L_Γ_ has several zero eigenvalues, one per connected component; each zero block of the global pair is the local pair (C_i_,A) itself, which is observable by assumption, so the conclusion is unchanged and connectivity is not required. □

**Remark** **1.** *Connectivity of* G *is not necessary for the result: if* G *is disconnected, the Laplacian has several zero eigenvalues, and the zero-eigenvalue blocks of the global pair are direct sums of the local pairs* (C_i_,A)*, each observable by assumption. The block construction in the proof covers every eigenvalue, zero or not, so Lemma 1 holds for arbitrary undirected graphs*.

The objective is to design, for each vehicle, a distributed observer whose residual $r_{i}=y_{i}-{\hat{y}}_{i}$ is simultaneously insensitive to d_i_ (*H*_∞_ requirement) and sensitive to f_s,i_ (*H*_−_ requirement), together with a threshold test on a windowed residual-energy functional that raises an alarm when J_er,i_(t) exceeds a calibrated threshold. The next section develops the design.

## 3. Coupling-Aware Detector Design

### 3.1. Distributed Observer

Throughout this section, the communication graph is assumed undirected and connected (Assumption 1): the modal decomposition of (7) and the LMI conditions of Theorems 1–3 rely on the symmetry of the Laplacian and do not extend to directed or switching topologies; see Section 5 for the extension path.

Vehicle i runs the observer

(4) $${\dot{\hat{x}}}_{i}(t)=A{\hat{x}}_{i}(t)+Bu_{i}(t)+L_{i}\left(y_{i}(t)-C{\hat{x}}_{i}(t)\right)+\sum_{j\neq i}\Gamma_{\mathrm{ij}}H({\hat{x}}_{j}(t)-{\hat{x}}_{i}(t))$$

with estimated output ${\hat{y}}_{i}=C{\hat{x}}_{i}$ and a constant gain L_i_ ∈ ℝ^3×2^ to be designed. Two features distinguish (4) from a bank of standalone observers. First, the correction uses the local residual only, so no raw sensor data crosses the network; second, the coupling term $\sum \Gamma_{\mathrm{ij}}$ uses the same H as the plant, which turns the drag coupling from a source of unmodelled interaction into additional information. Distributed observers of this family have been studied for generic interconnected systems [34]; here, the graph structure buys something more specific, namely an exact modal decomposition. In (4), the coupling correction appears as the sum of $H({\hat{x}}_{j}-{\hat{x}}_{i})$ over the neighbours, mirroring the plant term of (1) exactly; the $-{\hat{x}}_{i}$ part is essential, since without it the error dynamics (5) would not follow.

Define the estimation error $e_{i}=x_{i}-{\hat{x}}_{i}$. Subtracting (4) from (1) gives

(5) $${\dot{e}}_{i}(t)=(A-L_{i}C)e_{i}(t)+D_{d}d_{i}(t)-L_{i}Ef_{s,i}(t)+\sum_{j\neq i}\Gamma_{\mathrm{ij}}H\left(e_{j}(t)-e_{i}(t)\right)$$

and the residual available to vehicle i is

(6) $$r_{i}(t)=y_{i}(t)-{\hat{y}}_{i}(t)=Ce_{i}(t)+Ef_{s,i}(t)$$

A common gain L_i_ = L for all i is sought; this keeps the design symmetric, simplifies the distributed implementation, and is what the theorems below deliver. Stacking $e={\left[\begin{array}{c} e_{1}^{T},\ldots ,e_{N}^{T} \end{array}\right]}^{T}$, d, and f_s_ similarly, the global error dynamics are

(7) $$\dot{e}(t)=\left(I_{N}⊗(A-\mathrm{LC})-L_{\Gamma }⊗H\right)e(t)+\left(I_{N}⊗D_{d}\right)d(t)-\left(I_{N}⊗\mathrm{LE}\right)f_{s}(t)$$

with r(t) = (I_N_ ⊗ C)e(t) + (I_N_ ⊗ E)f_s_(t). Let L_Γ_ = UΛU^T^ be the spectral decomposition with eigenvalues λ_0_ = 0 < λ_1_ ≤ … ≤ λ_N−1_ and orthogonal U. Under the modal change of coordinates e^(k)^ = (U^T^ ⊗ I_3_)e (and likewise for d,f_s_,r), (7) separates into N third-order systems:

(8) $${\dot{e}}^{\left(k\right)}(t)=(A-\mathrm{LC}-\lambda_{k}H)e^{\left(k\right)}(t)+D_{d}d^{\left(k\right)}(t)-\mathrm{LE}f_{s}^{\left(k\right)}(t),\;k=0,1,\ldots ,N-1$$

Because U is orthogonal, Parseval’s identity holds in time: $\sum_{i}\int ∥r_{i}{∥}^{2}\mathrm{dt}=\int \sum_{k}∥{\;r}^{\left(k\right)}{∥}^{2}\mathrm{dt}$, and likewise for d and f_s_. Performance specifications on the platoon therefore translate mode by mode, which is what makes the common-gain design tractable.

### 3.2. H_∞_ Robustness Condition

With f_s_ = 0, the design goal is to attenuate the disturbance-to-residual channel: residuals should stay quiet when no attack is present, since every false excursion of the residual pushes the false-alarm rate up. The performance index is

(9) $$\int_{0}^{\infty }∥\;r_{i}(t){∥}^{2}\mathrm{dt}<\gamma^{2}\int_{0}^{\infty }∥\;d_{i}(t){∥}^{2}\mathrm{dt}$$

for zero initial error. The bound ν on the observer gain announced in the Introduction appears next; it is part of the statement, not an afterthought, because without it the infimum of γ over L is zero (an arbitrarily fast observer drives the residual norm to zero at every frequency, at the price of an unimplementable gain and noise amplification). The constant ν is chosen a priori from sampling-rate and actuator-rate considerations.

**Theorem** **1.** *Given γ > 0 and a gain bound ν > 0, suppose there exist P ≻ 0 and W ∈ ℝ^3×2^ with σ_max_(W) ≤ ν such that, for every eigenvalue λ_k_ of L_Γ_,*

(10) $$\begin{array}{r} \Xi_{k}:=\left[\begin{array}{cc} \mathrm{He}\left\{P(A-\lambda_{k}H)-\mathrm{WC}\right\}+C^{T}C & PD_{d}\\ D_{d}^{T}P & -\gamma^{2}I \end{array}\right]≺0 \end{array}$$

*Then, L = P^−1^W renders the error system (7) asymptotically stable and guarantees (9)*.

**Proof of Theorem** **1.** Apply the modal change of coordinates to (7) with f_s_ = 0. By Parseval’s identity, (9) holds if every modal system

$${\dot{e}}^{\left(k\right)}=(A-\mathrm{LC}-\lambda_{k}H)e^{\left(k\right)}+D_{d}d^{\left(k\right)},\;r^{\left(k\right)}=Ce^{\left(k\right)}$$

satisfies the same inequality. For mode k, use the storage function V_k_ = e^(k)T^Pe^(k)^. Since W = PL,

$${\dot{V}}_{k}=e^{(k)T}\mathrm{He}\left\{P(A-\lambda_{k}H)-\mathrm{WC}\right\}e^{\left(k\right)}+2e^{(k)T}PD_{d}d^{\left(k\right)}.$$

Adding r^(k)T^r^(k)^ − γ^2^d^(k)T^d^(k)^ = e^(k)T^C^T^Ce^(k)^ − γ^2^d^(k)T^d^(k)^ gives exactly

$${\left[\begin{array}{c} e^{\left(k\right)}\\ d^{\left(k\right)} \end{array}\right]}^{T}\Xi_{k}\left[\begin{array}{c} e^{\left(k\right)}\\ d^{\left(k\right)} \end{array}\right]<0$$

by (10). Hence, ${\dot{V}}_{k}+∥r^{\left(k\right)}{∥}^{2}-\gamma^{2}∥d^{\left(k\right)}{∥}^{2}<0$. Integrating from 0 to ∞ with V_k_(0) = 0 and V_k_(∞) ≥ 0 yields the modal version of (9); summing over k and using Parseval gives (9). Stability follows from the same inequality with d^(k)^ = 0, since then ${\dot{V}}_{k}<-∥r^{\left(k\right)}{∥}^{2}\leq 0$ and (C,A − λ_k_H) is observable. □

### 3.3. H_−_ Sensitivity Condition

With d = 0, the complementary requirement is that the residual carries enough energy about the attack. The index is

(11) $$\int_{0}^{\infty }∥\;r_{i}(t){∥}^{2}\mathrm{dt}>\eta^{2}\int_{0}^{\infty }∥\;f_{s,i}(t){∥}^{2}\mathrm{dt}$$

Again, by Parseval, it suffices to enforce (11) per mode with $r^{\left(k\right)}=Ce^{\left(k\right)}+Ef_{s}^{\left(k\right)}$.

**Remark** **2.** *Indices (9) and (11) are platoon-level statements: Parseval’s identity bounds the aggregate residual energy summed over vehicles. Because the modal transformation is orthogonal and the LMIs of Theorems 1 and 2 hold for every mode with the common constants* γ *and* η*, the same two constants certify each individual channel: the residual energy induced in vehicle* i *by its own disturbance is bounded by* γ^2^ *times the disturbance energy, and the attack energy visible in* r_i_ *is at least* η^2^ *times the attack energy. The per-vehicle test of Section 3.6 therefore inherits the certified levels vehicle by vehicle, which is what allows per-vehicle thresholds in Section 4.*

**Theorem** **2.** *Given η > 0 and the same gain bound ν, suppose there exist P ≻ 0 and W ∈ ℝ^3×2^ with σ_max_(W) ≤ ν such that, for every λ_k_,*

(12) $$\begin{array}{r} \Psi_{k}:=\left[\begin{array}{cc} \mathrm{He}\left\{P(A-\lambda_{k}H)-\mathrm{WC}\right\}-C^{T}C & -\mathrm{WE}-C^{T}E\\ {-E}^{T}W^{T}-E^{T}C & (\eta^{2}-1)I \end{array}\right]≺0 \end{array}$$

*Then, L = P^−1^W guarantees (11), provided that the gain renders every modal matrix A−LC−λ_k_H Hurwitz. This stability hypothesis is stated explicitly because condition (12) alone does not imply it (see the proof); in the joint design of Theorem 3 it is supplied by (10)*.

**Proof of Theorem** **2.** For mode k with d^(k)^ = 0, expand the residual norm:

$$∥\;r^{\left(k\right)}{∥}^{2}=e^{(k)T}C^{T}Ce^{\left(k\right)}+2e^{(k)T}C^{T}Ef_{s}^{\left(k\right)}+f_{s}^{(k)T}E^{T}Ef_{s}^{\left(k\right)},\;E^{T}E=I.$$

With ${\overset{‾}{V}}_{k}=e^{(k)T}Pe^{\left(k\right)}$ and W = PL, the quantity

$${\dot{\overset{‾}{V}}}_{k}-∥r^{\left(k\right)}{∥}^{2}+\eta^{2}∥f_{s}^{\left(k\right)}{\;∥}^{2}$$

equals ${\left\lceil \begin{array}{c} e^{\left(k\right)}\\ f_{s}^{\left(k\right)} \end{array}\right\rceil }^{T}\Psi_{k}\left\lceil \begin{array}{c} e^{\left(k\right)}\\ f_{s}^{\left(k\right)} \end{array}\right\rceil$, which is negative by (12). Integrating from 0 to ∞ with ${\overset{‾}{V}}_{k}(0)=0$ gives the modal form of (11); summing over k gives (11). With f_s_(k) = 0, (12) reduces to ${\dot{V}}_{k}<∥r(k){\;∥}^{2}$, which does not imply stability: its (1,1) block gives He{PA_k_} ≺ C^T^C − (1 − η^2^)^−1^(WE + C^T^E)(WE + C^T^E)^T^, and the right-hand side, the difference of two positive semidefinite matrices, is not negative definite, so He{PA_k_} ≺ 0 does not follow from (12) alone. Standalone stability is therefore an explicit hypothesis of Theorem 2. In the joint design of Theorem 3 it holds automatically: condition (10) with d(k) = 0 gives ${\dot{V}}_{k}<-∥r(k){∥}^{2}\leq 0$, and asymptotic stability follows from the observability of (C,A − λ_k_H), exactly as in the proof of Theorem 1. □

**Remark** **3.** *Because* E *has orthonormal columns here,* r_i_ *contains the attack directly: when* e_i_ ≈ 0*,* ∥ r_i_ ∥=∥ f_s,i_ ∥*. Letting the attack window shrink then forces* η ≤ 1 *in (11), whatever the observer. The ceiling is a property of the attack channel, not of the LMI. Values* η < 1 *quantify how much of the attack energy survives in the residual at the detector input after the transient decays*.

### 3.4. Joint Design

**Theorem** **3 (joint performance).** *Given γ > 0 and η > 0, if a single pair (P,W) with P ≻ 0 and σ_max_(W) ≤ ν satisfies both (10) and (12) for every λ_k_, then the observer gain L = P^−1^W meets (9) and (11) simultaneously*.

**Proof of Theorem** **3.** The gain L satisfies the hypothesis of Theorem 1 with (P,W) and of Theorem 2 with $\left(\overset{‾}{P},\overset{‾}{W}):=(P,W\right)$; both conclusions then hold for the same error system. The stability hypothesis of Theorem 2 is met because the same pair (P, W) satisfies (10), which supplies asymptotic stability, as shown in the proof of Theorem 1. □

A note on what is certified: the LMI conditions of Theorems 1–3 are sufficient but not necessary, and the scalars γ and η are prescribed design targets certified by these conditions, not necessarily the achieved closed-loop indices of (9) and (11); see Remark 4 in Section 4.1 for the realised values of the printed gains.

Two remarks are in order. First, the sign pattern of (10) and (12) differs exactly where it should: the +C^T^C block rewards residual energy in (10) as the quantity being attenuated, while the −C^T^C block in (12) penalises it, because in the *H*_−_ inequality the residual is the subject. The conflicting signs are why a single inequality cannot express both requirements, and why a common Lyapunov matrix is a constraint rather than a convenience. Second, with E^T^E = I the (2,2) block of (12) is negative for η < 1, so the sensitivity LMI stays well conditioned in the useful range of η.

### 3.5. Gain Computation

The bisection that returns the joint gain is summarised in Algorithm 1.
**Algorithm 1:** Joint *H*_−_/*H*_∞_ observer gain.Step 1:Choose $\overset{‾}{\eta }$ and a gain bound ν; set $\gamma_{\mathrm{lo}}^{2}=0$, $\gamma_{\mathrm{hi}}^{2}=\gamma_{u}^{2}$ (any known upper bound, e.g., from the *H*_∞_-only design), and tolerance ε > 0.
Step 2:Set $\gamma^{2}=(\gamma_{\mathrm{lo}}^{2}+\gamma_{\mathrm{hi}}^{2})/2$ and test feasibility of (10)-(12) with η held at the value chosen in Step 1, over variables P ≻ 0, W, subject to P ≽ I and σ_max_(W) ≤ ν. If feasible, set $\gamma_{\mathrm{hi}}^{2}=\gamma^{2}$; otherwise set $\gamma_{\mathrm{lo}}^{2}=\gamma^{2}$. Every feasibility answer is verified by direct eigenvalue inspection of (10) and (12) at the returned (P, W); the reported levels below are the smallest eigenvalue-verified ones, which removes solver-tolerance ambiguity at the feasibility boundary.
Step 3:Repeat Step 2 until $\gamma_{\mathrm{hi}}^{2}-\gamma_{\mathrm{lo}}^{2}\leq \varepsilon$. Return L = P^−1^W at the last feasible iterate.

Two implementation notes: The normalisation P ≽ I is a numerical safeguard; the correct homogeneity statement is that the inequalities are homogeneous in (P,W), γ^2^, and η^2^ jointly: scaling both by α > 0 rescales γ^2^ and η^2^ by the same factor, so one may fix the normalisation P ≽ I without loss of generality. The constraint σ_max_(W) ≤ ν is an LMI W, namely $\left[\begin{array}{cc} \nu I & W\\ W^{T} & \nu I \end{array}\right]≽0$ after rescaling, and removing it makes the γ-infimum trivial as discussed. Bisection on a scalar with linear-rate convergence replaces the BMI alternation used in early mixed-norm designs; global BMI solvers [35] remain an option when the gain bound is dropped. Two further clarifications: First, the bound constrains σ_max_(W) = σ_max_(PL) rather than σ_max_(L) itself; since σ_max_(L) ≤ σ_max_(PL)/σ_min_(P), a bound on the latter follows whenever σ_min_(P) is bounded away from zero, which the normalisation P ≽ I ensures. Second, for λ = 0 the uncorrected modal matrix A − λH = A is not Hurwitz (it contains the double integrator), so stability of the error dynamics is conferred entirely by the correction term −LC; this is exactly what the negative-definite upper-left blocks of (10) and (12) certify. Regarding conservatism, the homogeneity argument shows that the normalisation P ≽ I itself costs no feasibility; any conservatism enters only indirectly, through the route from the bound on W to a bound on L, whose tightness depends on σ_min_(P) and is not quantified here; when a hard implementation budget concerns L itself, ν should be treated as a tuning parameter rather than a strict certificate on L.

### 3.6. Residual Evaluation and Decision Logic

Instantaneous residuals are noisy decision variables; the test statistic is the windowed energy

(13) $$J_{\mathrm{er},i}(t)=\frac{1}{T}\int_{t-T}^{t}∥\;r_{i}(\tau ){∥}^{2}d\tau$$

with window T = 5 s. Thresholds are calibrated offline on attack-free data: simulate an ensemble of N_c_ attack-free runs spanning the expected spread of initial estimation errors and disturbance levels, pool the samples of J_er,i_ taken after an initial settling horizon, and take the empirical (1 − α)-quantile,

J_th,i_ = Q_1−α_(J_er,i_) (14)

with α = 0.005 and N_c_ = 100 in this study. A mean-plus-κ rule would reduce to the same number under Gaussian residuals, but the manoeuvre transients of Section 4 leave the residual energy heavy-tailed, and the quantile form holds the false-alarm rate at its nominal level regardless of the tail shape. The decision rule is

J_er,i_(t) > J_th,i_ ⇒ alarm on vehicle i; otherwise no alarm (15)

**Figure 2:**
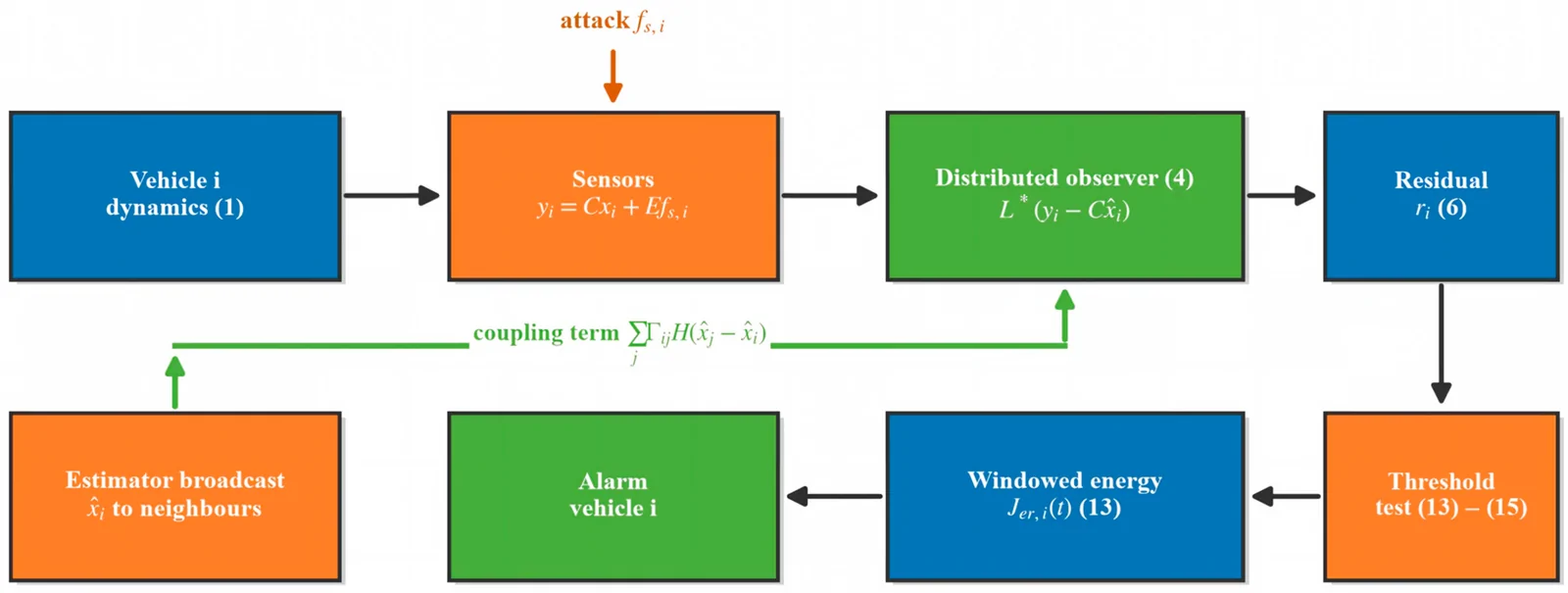
Detection framework. Each vehicle runs a local observer whose correction mixes its own sensor residual with the estimates broadcast by its neighbours; the windowed residual energy is tested against a per-vehicle threshold calibrated offline, and an alarm switches the local controller to a conservative schedule.

For the DoS case, the residual itself is still computable while the link is down (vehicle i keeps predicting the neighbour state open loop, exactly as in (4) with the coupling term fed by the prediction), so the same rule applies without modification. The detector reports an anomaly, not an attack type: a genuine hard manoeuvre by a neighbour produces a qualitatively similar prediction mismatch, and Section 4.4 quantifies this specificity limit. The connection between these indices and the practical detector is worth stating. The *H*_∞_ level γ bounds the disturbance energy that can reach the residual, so the empirical-quantile threshold of (14) holds the false-alarm rate at its nominal level α whenever the disturbance stays within the calibration envelope. The *H*_−_ level η guarantees a residual-energy floor of η^2^ times the attack energy over the window, so the windowed statistic exceeds the threshold with a probability that grows with the attack energy and the window length, and decreases with γ and the threshold. Detection probability is therefore governed by the ratio of certified attack energy to threshold energy; Section 4.5 quantifies the threshold side of this trade-off, and the finite-window caveat is discussed in Section 5. The detection framework described in this section is summarised in Figure 2.

## 4. Simulation Study

### 4.1. Setup

The platoon consists of N = 5 vehicles in a bidirectional chain, so Γ_ij_ = 1 for |i − j| = 1 and zero otherwise; the Laplacian eigenvalues are {0,0.38,1.38,2.62,3.62}. The vehicle parameters are σ = 0.5 (engine time constant 2 s), disturbance weights ϑ_1_ = 0.1, ϑ_2_ = 0.2, and coupling coefficient 𝓁 = 0.1. The platoon is controlled by a CACC law with spacing feedback gains k_1_ = 0.35, k_2_ = 0.90, feedforward gain k_3_ = 0.80, own-acceleration damping k_4_ = 1.00, and desired spacing d_0_ = 10 m; the leader tracks a 15 m/s cruise profile with velocity feedback 0.60, accelerates at 1.2 m/s^2^ during t ∈ [10,20] s, and brakes at the same level during t ∈ [50,60] s. The vehicles start at the desired spacing, so the platoon is at equilibrium before any attack begins. Each vehicle is disturbed by d_i_(t) = 0.1sin(0.4t + ϕ_i_) with independent phases. All simulations use Euler integration with step 0.02 s over 100 s. Table 1 collects the parameter values used in all runs.

The follower CACC law is

$$u_{i}(t)=k_{1}e_{i}(t)+k_{2}{\dot{e}}_{i}(t)+k_{3}a_{i-1}(t)-k_{4}a_{i}(t)$$

with spacing error

e_i_(t) = (p_i−1_(t) − p_i_(t)) − d_0_,

and the leader runs

u_1_(t) = 0.60 ⋅ (v_ref_(t) − v_1_(t));

a_i−1_ is the preceding vehicle’s acceleration from the V2V feedforward channel and a_i_ the vehicle’s own acceleration.

The observer design of Section 3 uses gain bound ν = 5 and target sensitivity $\overset{‾}{\eta }=0.9$. Algorithm 1 returns γ^*^ = 0.27 and gain

$$\begin{array}{r} L^{*}=\left[\begin{array}{cc} 1.3698 & -0.0732\\ 3.2769 & -0.2907\\ 0.4879 & 0.0671 \end{array}\right] \end{array}$$

**Figure 3:**
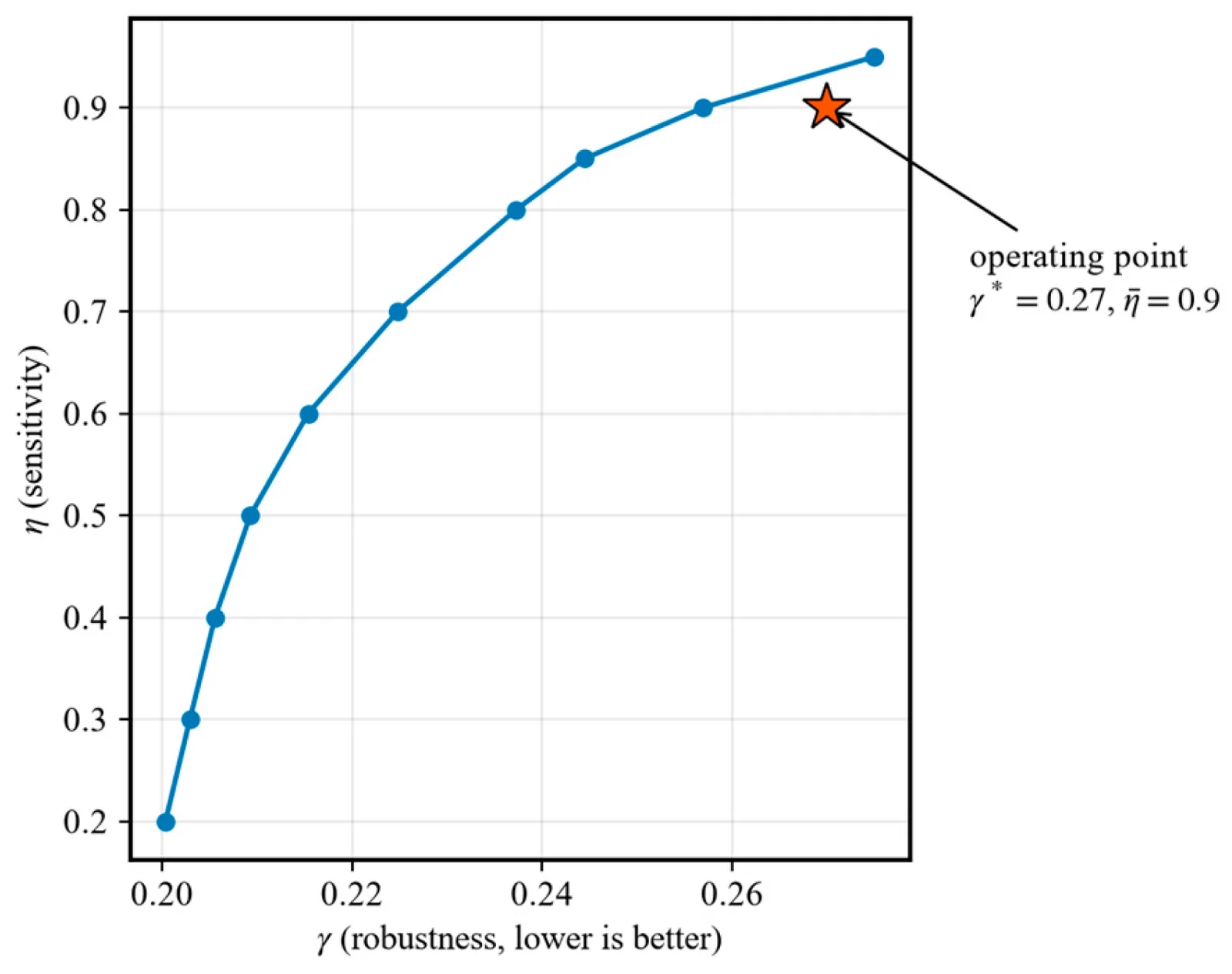
Pareto frontier between the *H*_∞_ robustness level γ and the *H*_−_ sensitivity level η for the five-vehicle platoon, traced by the bisection of Algorithm 1 at several $\overset{‾}{\eta }$ values; the star marks the operating point ($\overset{‾}{\eta }=0.9$).

For comparison, an *H*_∞_-only design (Theorem 1 alone) gives γ = 0.0622 with the sensitivity constraint (12) dropped at the same gain bound, i.e., it buys the best robustness but certifies no sensitivity lower bound. The Pareto curve of γ against η, traced by rerunning the bisection at several $\overset{‾}{\eta }$ values, is shown in Figure 3. This baseline is the robustness-optimal design point of the same LMI family (Theorem 1) and is the standard comparator in the mixed-norm literature [18]; its near-zero sensitivity is a property of the *H*_∞_-optimal point itself, not of deliberately skewed tuning. The certified pair (γ, η) = (0.27, 0.90) holds at the printed (rounded) gain with strictly negative margins on both LMIs, verified by direct eigenvalue inspection as prescribed in Algorithm 1. Table 2 lists the certified levels and gains of both designs.

The thresholds follow (14) with N_c_ = 100 attack-free runs, whose initial estimation errors and disturbance amplitudes are randomised; on an independent set of 100 attack-free runs they hold the per-sample false-alarm rate to 0.46% ± 0.26 percentage points after t = 10 s. The resulting per-vehicle thresholds are J_th_ = [1.11 × 10^−3^, 9.43 × 10^−4^, 9.59 × 10^−4^, 9.25 × 10^−4^, 1.04 × 10^−3^].

**Remark** **4.** η *and* γ *are the design targets certified by (12) and (10), not the achieved indices of (11) and (9). At the printed (rounded) gains, frequency-swept over the full 15-dimensional error system, the realised H*_∞_ *norms are 0.267 (proposed) and 0.0622 (baseline), and the achieved H*_−_ *indices of (11) are 0.97 (proposed) and 0.65 (baseline): the feedthrough E in (3) passes the attack directly to the residual, which is why both indices sit near the ceiling of Remark 3, while the observer-dependent state-channel sensitivities (feedthrough removed) are of order* 3 × 10^−6^* and* 3 × 10^−5^*, respectively. The practical content of the H*_−_ *guarantee is an energy floor: at least *η^2^* of the attack energy reaches the residual. It is not a per-sample detection probability; the windowed test of Section 3.6 converts the floor into detection only when the attack energy collected over the window exceeds the threshold, which is precisely the regime distinction between the transient attacks of Section 4.3 and the zero-dynamics attacks of Section 4.4*.

### 4.2. State Estimation Under Attack-Free Conditions

**Figure 4:**
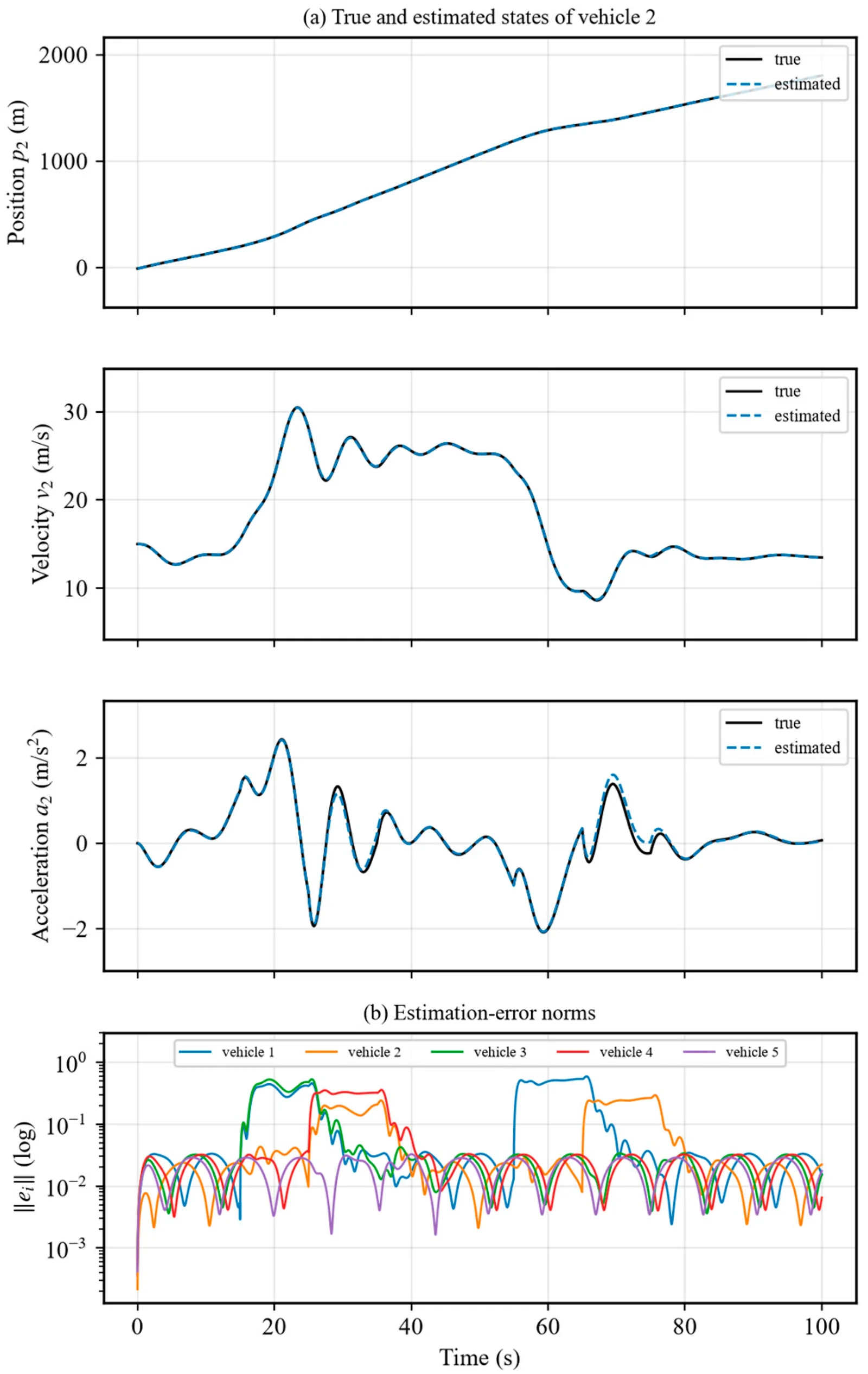
(**a**) True (solid) and estimated (dashed) position, velocity, and acceleration of vehicle 2. (**b**) Estimation-error norms of all five vehicles (log scale).

Figure 4a shows the true and estimated states of vehicle 2 over the full run; the estimate tracks the manoeuvre at t = 10 − 20 s, with a transient that dies out within roughly 2 s. Figure 4b plots the estimation-error norm of all five vehicles on a logarithmic scale: errors decay by two orders of magnitude within the first 10 s and remain there, which confirms that the closed-loop observer is stable under the CACC input and the sinusoidal disturbances, as Theorem 3 predicts. The windowed residual energies in attack-free operation sit below the calibrated thresholds except in short bursts around the two manoeuvre transients, where the platoon state changes fastest; those bursts, rather than the noise floor, set the threshold level.

### 4.3. Transient Attack Detection

Transient FDI attacks are injected into the velocity channels of vehicles 1–4 according to: vehicle 1, 3sin(0.6t) on t ∈ [15,25] s and a +3 m/s step on t ∈ [55,65] s; vehicle 2, 1.5sin(0.6t) on [25,35] s and a +1.5 m/s step on [65,75] s; vehicle 3, 3.5sin(0.6t) on [15,25] s; vehicle 4, a +2 m/s step on [25,35] s. Vehicle 5 is not attacked and serves as a false-alarm control.

**Figure 5:**
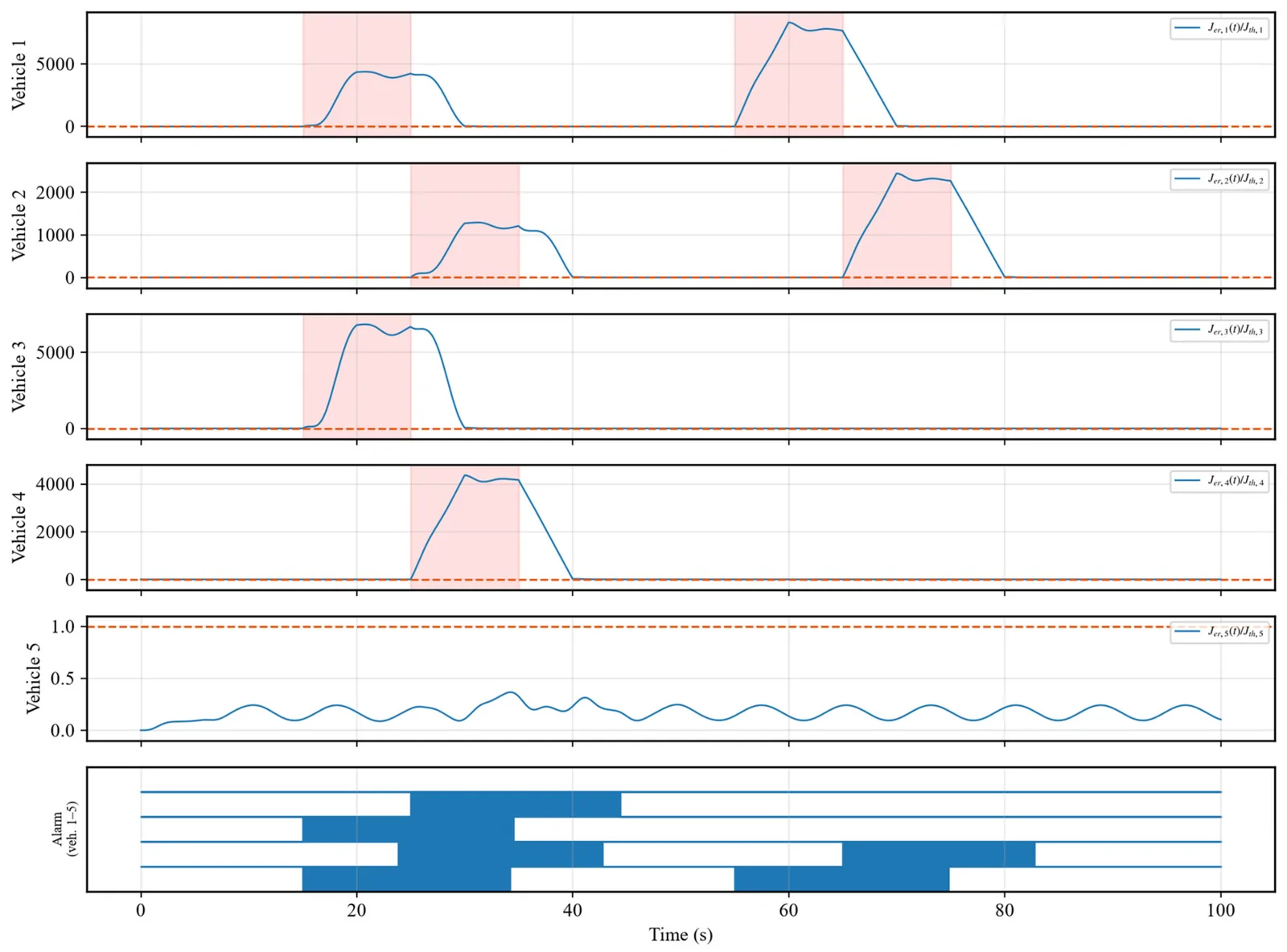
Windowed residual energy J_er,i_ (solid) and threshold J_th,i_ (dashed) for vehicles 1–5 under transient attacks; attack windows are shaded.

**Figure 6:**
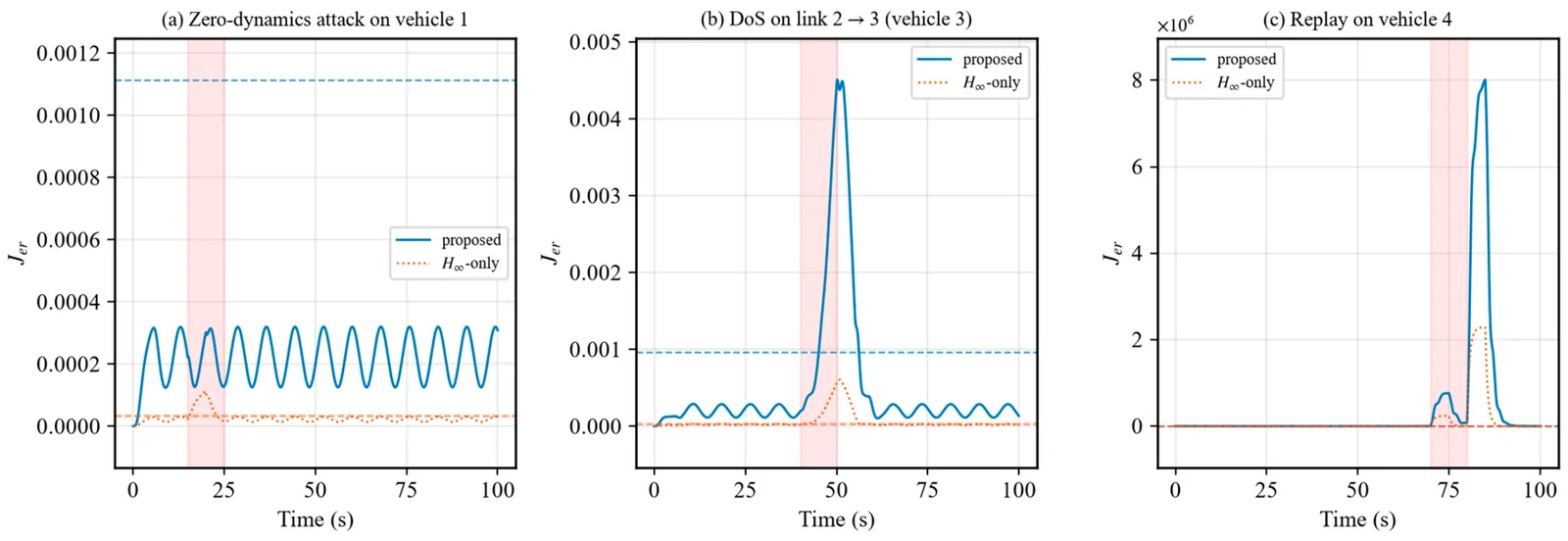
Windowed residual energies under stealthy attacks: (**a**) zero-dynamics attack on vehicle 1; (**b**) DoS on the link 2 → 3; (**c**) replay on vehicle 4. Proposed observer (solid) versus *H*_∞_-only baseline (dotted); thresholds dashed. The shaded band marks the attack window.

Figure 5 overlays the windowed residual energy of each vehicle with its threshold and shows the binary detection trace derived from it; the windowed residual energies under the stealthy attacks of Section 4.4 are displayed in Figure 6. Every attack window is detected, with mean detection delays of 0.00 s for the proposed observer against 0.00 s for the *H*_∞_-only baseline. The detection rate (fraction of attacked samples above threshold within attack windows) is 100.0% for the proposed design and 100.0% for the baseline, while the false-alarm rate measured on the independent attack-free ensemble is 0.46% for the proposed design versus 0.52% for the baseline. Vehicle 5 stays quiet throughout, as it should.

### 4.4. Stealthy and Advanced Attacks

Three harder cases follow. For the zero-dynamics attack, vehicle 1 is driven through its one-dimensional zero dynamics: the internal state g obeys g(t) = −1.5g(t) with g(15) = 0.1 and the injected signal is f_1_(t) = 0.3·g(t) (peak amplitude 0.03 m/s); the attack signal itself decays with rate 1.5, so almost all of its energy is gone within 3 s. Such attacks are known to be poorly visible to norm-based monitors [36], and the security-index literature [37,38] explains why: the attack excites precisely the least observable directions. Figure 6a compares the residual energies of the two observers on vehicle 1. Both residuals respond to the attack, with an integrated squared residual over the attack window of 2.15 × 10^−3^ for the proposed design versus 6.41 × 10^−4^ for the baseline, a factor of 3.35; the windowed test flags 0.0% of the attacked samples for the proposed design and 79.2% for the baseline. The higher sample count of the baseline on this particular attack is a threshold-scale effect, not a sensitivity advantage: its residual is quieter in nominal operation (γ = 0.0622 against γ = 0.27), so its calibrated threshold sits lower in absolute terms. Which realisation of a stealthy attack each observer flags more of is thus scenario- and threshold-dependent. The proposed design flags none of the attacked samples in this configuration because its calibrated threshold sits above the residual energy this attack can deliver within the window: the energy-floor reading of Remark 4 in its sharpest form. The absolute rates are further reduced because a 5 s evaluation window dilutes an attack whose own energy is concentrated in the first 3 s. A sliding-mode secure observer in the spirit of [39] was also implemented as an additional baseline (Table 3); its false-alarm rate reflects the standard literature operating point, discussed in Section 4.6. Two sensitivities are reported to avoid over-reading a single realisation. First, the window length: shortening the evaluation window to one second lowers the flagged fraction of the baseline to 57.2%, while the proposed observer still flags none of the attacked samples. Second, the attack parameters: Table 4 sweeps the peak amplitude (0.03–1.0 m/s), decay rate (0.5–3.0 s^−1^), and injection vehicle (1–3) over three randomised realisations each. Detection is strongly energy-dependent: at the documented peak amplitude of 0.03 m/s, no configuration is detected at all; at 0.3 m/s the detection rate rises to 52–79%, and at 1.0 m/s to 58–98%, with slower-decaying (longer-lived) attacks detected more often at every amplitude. Stealth is therefore a matter of attack energy rather than of injection location, consistent with the energy-floor reading of Remark 4 and with the fundamental limits of norm-based monitors identified in [36,37].

For the DoS attack, the link from vehicle 2 to vehicle 3 is blocked during t ∈ [40,50] s and vehicle 3 predicts vehicle 2 open loop (Section 3.6). Figure 6b shows the residual energy of vehicle 3. The proposed observer detects 48.8% of the window against 84.4% for the baseline. One specificity caveat is worth reporting: when vehicle 2 performs a genuine hard acceleration (a_2_ = 5 m/s^2^ for 3 s) instead of being jammed, vehicle 3’s prediction also degrades and an alarm fires on 77.4% of that interval (mean over 30 randomised runs). The detector responds to inconsistency, not to maliciousness, echoing the conclusions of DoS-specific platoon controllers [29]; type attribution would need side information that this paper does not assume. The proposed design’s gentler correction lets the prediction mismatch accumulate more slowly in the windowed statistic; what the certificate buys instead is the guaranteed sensitivity floor of Section 3.3, which the baseline lacks.

For the replay attack, vehicle 4’s measurements during t ∈ [70,80] s are replaced by its own t − 60 s data. The proposed observer flags 100.0% of the window (baseline: 100.0%). Replay detection rates are high because the platoon’s CACC state evolves measurably over the 5 s evaluation window, making stale data inconsistent with the neighbours’ estimates; dedicated watermarking schemes [30,31] would raise the bar for the attacker at the cost of extra signalling.

### 4.5. Monte Carlo Evaluation and ROC Analysis

Three rates are used consistently in this section and deserve distinct definitions. The false-alarm rate (FAR) is the fraction of samples above threshold on the independent attack-free validation ensemble. The outside-window exceedance rate is the fraction of samples above threshold outside the attack intervals during attacked runs; it is dominated by residual ringing while the estimates re-converge after the manoeuvres and attack onsets, and it is not a false-alarm rate in the operational sense. The DR is the fraction of attacked samples above threshold. The 0.46% FAR and the 10.55% exceedance rate therefore describe different ensembles and are not contradictory. Monte Carlo variability is reported as standard deviations (±1.2 percentage points on the detection rate and ±0.68 percentage points on the exceedance rate). Calibration used 100 attack-free runs and validation an independent set, so no calibration/test leakage arises.

**Figure 7:**
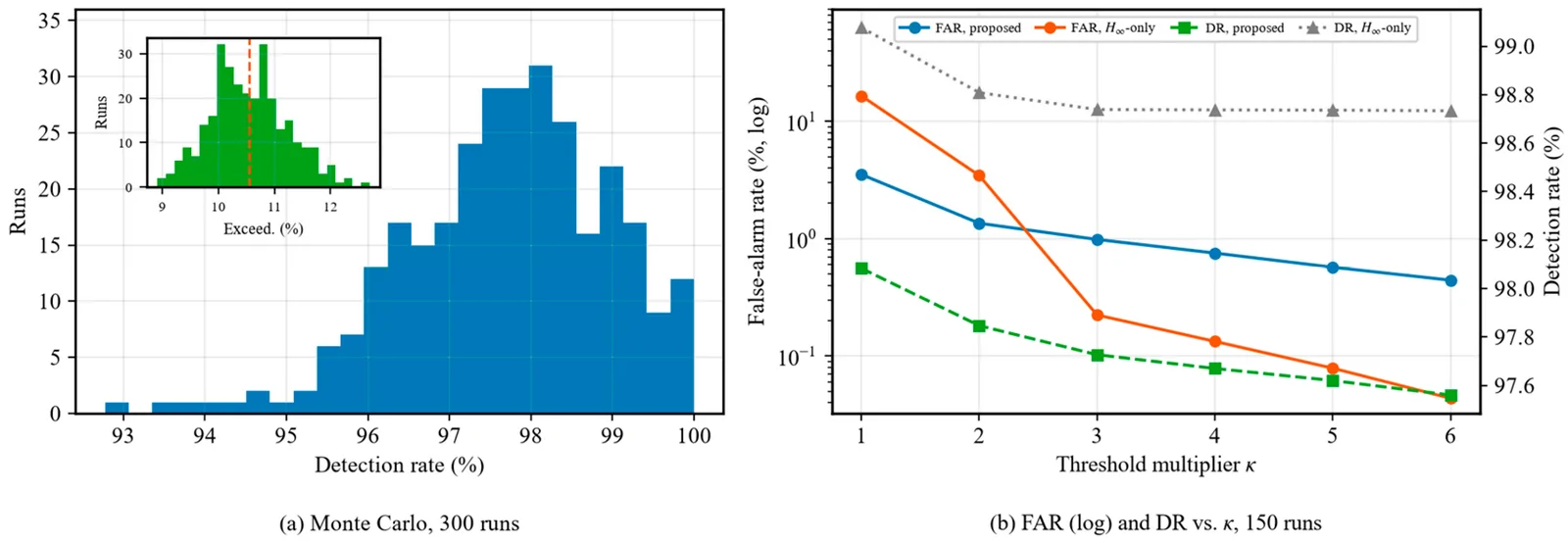
(**a**) Histograms of detection rate and of the outside-window exceedance rate over 300 Monte Carlo runs. (**b**) False-alarm rate (log scale, left axis) and detection rate (right axis) against the threshold multiplier κ; each run self-calibrates on its own attack-free segment. In the inset of (**a**), the dashed line marks the mean outside-window exceedance rate over the 300 runs.

To probe the robustness of the conclusions to nuisance variability, 300 independent runs were performed with randomised disturbance amplitudes (±20% around 0.1), randomised initial estimation errors up to ±0.5 per state, and attack onsets jittered by ±1 s, all drawn from independent uniform distributions. The detection rate over the transient-attack windows averages 97.7%, with a standard deviation of 1.2 percentage points; the histograms are shown in Figure 7a, and the exceedance rate outside the attack intervals, which includes residual ringing while the estimate re-converges after each episode, is 10.55% ± 0.68. The detection-rate spread is narrow enough that the conclusions are not an artefact of a particular seed.

Figure 7b sweeps the threshold multiplier κ over 150 randomised runs, each run self-calibrating its threshold on its own attack-free segment, and plots the false-alarm rate on a logarithmic axis together with the detection rate. Under this calibration rule the comparison reverses relative to the quantile calibration of Section 4.3, but only from κ = 3 upward: at κ = 1 and κ = 2 the proposed observer’s false-alarm rate still sits far below the baseline’s (3.52% against 16.44%, and 1.35% against 3.45%), while at κ = 3 and beyond the order flips (0.98% against 0.22% at κ = 3, and 0.44% against 0.04% at κ = 6): the curves cross between κ = 2 and κ = 3 as the baseline’s tighter residual core starts to dominate the mean-plus-κσ rule. For κ ≥ 3 the heavier tail of the proposed residual is the price of the worst-case certificate (11) that the baseline does not hold, and the penalty remains modest on blatant attacks (detection rates 97.7% versus 98.7% at κ = 3, evaluated on jittered onsets). Under the quantile calibration of (14) used for the validated results, the two observers are essentially matched (0.46% versus 0.52%, Section 4.3); the κ sweep is therefore best read as quantifying what the certificate costs under an alternative, moment-based self-calibration rule, not as a defect of the detector at its deployed operating point.

### 4.6. Comparison and Ablation

Three ablations isolate the role of each design choice (Table 5). Removing the coupling term from the observer (H = 0, observer redesigned at the same $\overset{‾}{\eta }=0.9$), with thresholds recalibrated under the same protocol, leaves the large-signal transient detection rate essentially unchanged (98.3% against 100.0%), but DoS detection collapses under recalibration (0.0% versus 48.8%): the unmodelled drag coupling inflates the decoupled observer’s attack-free residual, the recalibrated thresholds rise by two orders of magnitude on average (about 109× per vehicle, ranging from 12.5× to 257×), and the prediction-mismatch signature is drowned. The zero-dynamics row (0.0% against 20.6%) is likewise a threshold-scale artifact rather than a sensitivity difference: the decoupled observer’s windowed statistic hovers at its recalibrated threshold even in attack-free runs, so those crossings do not represent attack visibility. Even at the nominal weak coupling (𝓁 = 0.1), the coupling term is what keeps the observer consistent with the plant and the thresholds tight. It also keeps the *H*_−_ and *H*_∞_ guarantees of Section 3 valid when coupling is not negligible, as in tightly drafted heavy-vehicle platoons, without costing performance when it is. Doubling the disturbance level without recalibrating the thresholds leaves the validated false-alarm rate at 16.43% (against 0.46% at the nominal level): the *H*_∞_ design attenuates the disturbance channel but cannot absorb a twofold level change on its own; the freeze-on-alarm adaptive threshold introduced at the end of this section repairs exactly this dependence. Scaling the platoon to N = 8 vehicles degrades performance only slightly (detection rate: 100.0%, false alarms: 0.47%), indicating that the common-gain design tolerates moderate growth in string length. The coupling term matters where aerodynamic coupling is strong. Re-solving the joint design at growing l gives certified levels γ* = 0.26 at l = 0.1, 0.35 at l = 0.5, and 0.96 at l = 0.7 (a boundary-hugging certificate whose strict LMI margin is about 5 × 10^−5^, so it marks the feasibility edge rather than a robust operating point), with feasibility lost beyond that point; the decoupled design (H = 0) achieves γ* = 0.24 at l = 0, but its gain can no longer be certified by (10)–(12) once l reaches 0.5, at any γ ≤ 1. The certificate gap is matched by an empirical one: at l = 0.5 the coupling-aware observer detects 100.0% of the transient-attack window against 80.6% for the decoupled observer, and at l = 1.0 the figures are 100.0% against 28.0%, at matched validated false-alarm rates of about 0.5%. The ablation above should therefore be read as evidence about the tested light-coupling regime, not as a claim that the coupling term is empirically useless. On scalability, the Laplacian spectrum of a chain widens as N grows (its largest eigenvalue approaches four), so the common-gain modal family faces tighter feasibility margins for long strings; the eight-vehicle ablation evidences moderate growth, and generalisation to commercial-scale platoons of ten or more vehicles remains to be demonstrated. All methods in Table 5 share the same residual-window statistic and calibration protocol (empirical quantiles of attack-free runs); the sliding-mode baseline retains the literature design of [14,39] with its standard triggering rule, so its 99.84% false-alarm rate reflects that operating point rather than a tuned one, and an operating-point-matched recalibration is future work.

The communication model of Section 4.1 idealises the V2V links; to probe the most consequential gap, independent Bernoulli packet loss was added to every link, with dropped neighbour packets replaced by an open-loop drift prediction of the neighbour estimate (the same mechanism used for DoS in Section 3.6). Table 6 reports, for per-link loss probabilities of 1% to 20%, the false-alarm rate with the nominally calibrated thresholds and with thresholds recalibrated at each loss level, together with the transient-attack detection rate. Up to 20% loss on every link, both quantities are essentially unchanged (false-alarm rate of 0.50% without recalibration, detection rate of 100.0%): at 50 Hz sampling, isolated single-step drops are absorbed by the drift prediction and the 5 s window. Sustained loss is a different regime: the DoS case of Section 4.4, and the binding calibration dependence runs through the disturbance level rather than the link, which the adaptive scheme of Table 7 addresses.

The thresholds of (14) are tied to the calibration envelope. As a first step beyond static calibration, a freeze-on-alarm adaptive threshold was implemented: each vehicle tracks its residual-energy baseline with an exponential filter (time constant 20 s) that freezes while an alarm is active and for 3 s afterwards, and alarms when the windowed statistic exceeds κ_a_ times the tracked baseline. With κ_a_ = 40 calibrated on attack-free data at the nominal false-alarm rate, Table 7 compares static and adaptive thresholds under disturbance-level mismatch: at ×1.5 the two are comparable (0.76% versus 0.81%), but at ×2 the static threshold’s false-alarm rate rises to 16.43% while the adaptive threshold holds it at 2.09%, at a detection-rate cost on transient attacks from 100.0% to 99.9%. The large multiplier mirrors the heavy-tailed windowed statistic, and the tracker assumes attacks are sparse enough that the frozen baseline is rarely contaminated; adaptive thresholds for correlated or frequent attacks remain open. All rows of Table 7 are evaluated on the same 30-run validation ensemble as the 2× row of Table 5, so the static and adaptive columns differ only in the threshold rule; the ×1.0 static value (0.53%) accordingly differs slightly from the 100-run validated false-alarm rate of Section 4.3.

### 4.7. Impact on Platoon Control

**Figure 8:**
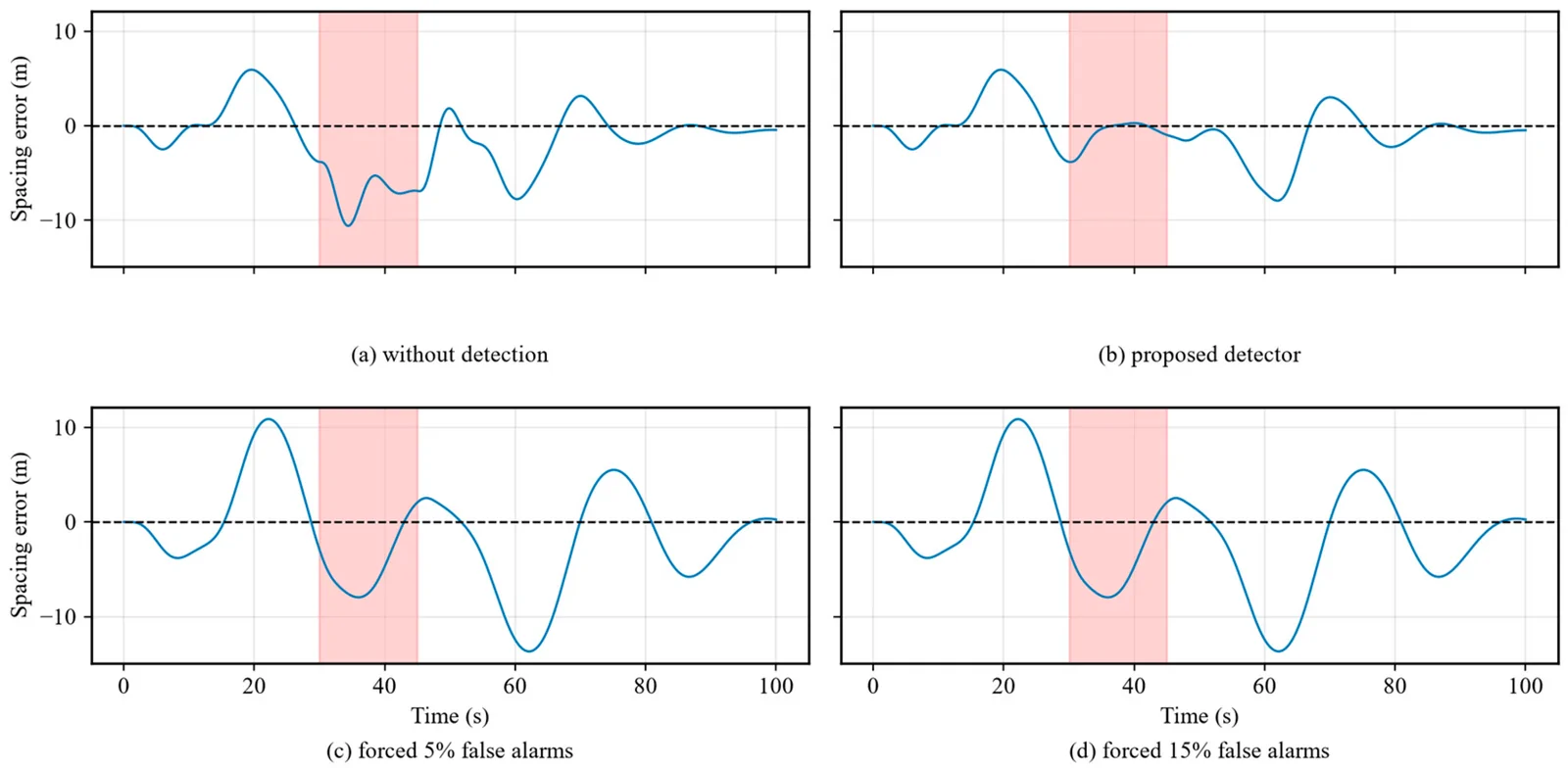
Spacing error of the follower pair under a +5 m/s sensor bias on vehicle 2: (**a**) without detection; (**b**) proposed detector; (**c**) forced 5% false alarms; (**d**) forced 15% false alarms. The light red band marks the attack window.

Detection is only useful if acting on it helps; attack-triggered reconfiguration for platoon control is studied from the control side in [40]. The detector was therefore embedded in the CACC loop: when vehicle i alarms, its controller switches to a conservative schedule (gains k_1_, k_2_ halved, estimates $\hat{x}$ used in place of the suspect measurement) and reverts once the residual stays below threshold for 3 consecutive seconds. A bias attack of +5 m/s on vehicle 2’s velocity sensor during t ∈ [30,45] s disturbs the loop. Figure 8a shows the spacing errors of the follower pair (2,3) under four regimes: no detection, the proposed detector, and two synthetic detectors whose false-alarm rates are forced to 5% and 15% by randomly injected alarms. Without detection the mean peak spacing error is 10.79 m; with the proposed detector it is 7.93 m; the 5% and 15% false-alarm groups reach 13.76 m and 13.76 m (means over 50 random seeds). The proposed detector cuts the mean peak error by about a quarter, but the two forced-alarm regimes do not share in the benefit: their peak errors exceed the no-detection case, because at a 5% or 15% per-sample false-alarm rate the 3 s release timer of the conservative schedule essentially never elapses, so the alarmed vehicle is latched in the conservative mode for the rest of the run. The forced regimes thus answer the question they were designed for: false alarms are not a benign tax on average performance but, beyond a modest rate, a qualitative regime change. The proposed detector triggers a single conservative switch per run on average, and the full safety suite is reported in Remark 5.

**Remark** **5.** *Peak spacing error alone is an incomplete safety metric, so the full suite is reported here (50 seeds per regime). Minimum inter-vehicle distance over all pairs: the attack produces collision-level overshoot in the worst seeds of every regime (worst values −1.20 m without detection, −1.64 m with the proposed detector, and −7.75 m/−7.75 m under forced 5%/15% false alarms), so the detector mitigates the hazard but does not eliminate it, and the latched conservative schedule drives the forced regimes deep into collision territory. Recovery time after the attack (|e*_23_*| below 0.5 m sustained for 3 s) averages 37.4 s with the proposed detector against 39.5 s without detection and 50.3 s/50.3 s under forced false alarms. The string-stability ratio (peak spacing error of pair (3,4) over that of pair (2,3)) averages 1.17 with the proposed detector against 0.92 without detection. The 10.55% outside-window exceedances of Section 4.5 concentrate around the manoeuvre transients; the forced-alarm regimes of Figure 8c,d show what a detector with such a sample-level false-alarm rate would cost in closed loop, and they are why the validated false-alarm rate, not the exceedance rate, is the quantity that matters once alarms drive controller switching. Two unfavourable directions deserve explicit note: in the worst seed the proposed detector’s minimum spacing (−1.64 m) is deeper than without detection (−1.20 m), with the conservative switching trading a smaller mean excursion against a deeper worst-seed undershoot, and the mean string-stability ratio of 1.17 exceeds the no-amplification value of one, so the mitigated excursion is still propagated downstream at a slightly amplified magnitude*.

## 5. Discussion

Three limitations should be stated plainly. First, the disturbance and attack models are energy-bounded, and the guarantees are input–output inequalities rather than hard detection probability bounds; the Monte Carlo study partially fills the gap but is simulation evidence. Second, the attack is assumed to enter a single known channel with E of full column rank; simultaneous sensor and actuator attacks with unknown E would require extending the sensitivity condition, for example, along the set-membership route taken in [22]; or augmenting the observer, as in attack-reconstruction schemes for platoons [23]. Third, the communication graph is static and undirected; directed switching topologies, as in trust-based monitoring [24], would break the modal decomposition and call for a common-Lyapunov or multiple-Lyapunov reformulation.

The scope of the guarantees deserves the same treatment. The bounds are energy based: for finite-duration, spatially localised attacks the *H*_−_ index bounds only the residual energy collected over the attack window, so short attacks yield proportionally little residual energy. This is precisely the zero-dynamics regime of Section 4.4, and it is why no norm-based monitor can claim reliable detection of arbitrarily stealthy attacks [36,37]; the detector should be composed with watermarking or physical-invariant checks when such adversaries are in scope. The communication layer remains partially idealised: Section 4.6 now evaluates independent packet loss on every link (Table 6), but delay, measurement noise, asynchronous sampling, varying link quality, and topology changes are not modelled. The *H*_∞_ channel partially covers such effects insofar as they act as additional disturbance inputs, but a dedicated evaluation on a realistic V2V communication model is left to future work. For directed or switching topologies the modal decomposition fails because the Laplacian is no longer symmetric; a common- or multiple-Lyapunov reformulation over the switching set would then be required, and we regard this as the main theoretical extension. Finally, because the thresholds of (14) are calibrated offline, they are tied to the disturbance and manoeuvre envelope of the calibration ensemble: operation outside that envelope without recalibration degrades the false-alarm rate, as the 16.43% entry of Table 5 illustrates; the freeze-on-alarm adaptive threshold of Section 4.6 (Table 7) is a first step towards online threshold adaptation and holds the false-alarm rate near its nominal level under a twofold disturbance mismatch.

On the engineering side, the window length T trades detection delay against false alarms: shorter windows react faster but raise the variance of J_er,i_ and hence the threshold. The choice T = 5 s was made on the validation data; operators with stricter delay budgets can shrink T at the cost of a higher threshold (or a larger multiplier κ). Computationally, the design is offline and small (ten 4 × 4 LMIs, one pair per Laplacian eigenvalue), and the online cost per vehicle is third-order integration plus a windowed sum, well within automotive micro-controller budgets. Finally, while the *H*_−_/*H*_∞_ pair dominates the *H*_∞_-only baseline here, detectors that watermark the sensor channel [30,31] or that exploit physical invariants across vehicles remain valuable as independent layers; the present scheme composes with them naturally because it consumes only the broadcast estimates that a platoon controller already exchanges. Moreover, the baselines of Section 4.6 are deliberately of the same residual-based family; detector families that exploit different information, namely sparse attack identification [37], secure-control design against resource-limited adversaries [36], watermarking-based replay defences [30,31], and the finite-time filter of [26], are complementary rather than directly comparable, and composing the present certificates with such layers is a promising direction.

## 6. Conclusions

This paper has developed a distributed attack-detection scheme for ICV platoons in which each vehicle estimates its own longitudinal state with an observer whose correction mixes the local residual with the state estimates broadcast by its neighbours. A modal decomposition over the platoon graph reduces the *H*_∞_ and *H*_−_ design conditions to one LMI family per Laplacian eigenvalue; a single observer gain satisfying both conditions is computed by bisection, with an explicit gain bound that removes the trivial high-gain optimum. On a five-vehicle platoon under transient, zero-dynamics, DoS, and replay attacks, the detector achieves a 97.7% mean detection rate on transient-attack windows across 300 Monte Carlo runs at a validated false-alarm rate of 0.46%; matches an *H*_∞_-only observer on transient and replay attacks while certifying a worst-case sensitivity the baseline lacks, at the cost of a lower detection rate on single-link denial-of-service attacks (48.8% against 84.4%), a trade-off stated plainly in Section 4.4; and, embedded in a CACC loop, cuts the mean peak spacing error during a sensor attack by about a quarter with a single conservative switch per run, while forced false-alarm rates of 5–15% latch the conservative schedule and degrade spacing beyond the no-detection case. Robustness to per-link packet loss up to 20% and a freeze-on-alarm adaptive threshold that holds the false-alarm rate near nominal under a twofold disturbance mismatch are demonstrated in Section 4.6. Future work will address directed and switching topologies, joint sensor–actuator attacks with unknown injection matrices, and a systematic evaluation at stronger aerodynamic coupling (e.g., tightly drafted heavy-vehicle platoons).

## Figures and Tables

**Table 1:** Simulation parameters.

Parameter	Symbol	Value
Number of vehicles	N	5 (chain); 8 in scalability ablation
Engine time constant	1/σ	2 s (σ = 0.5)
Disturbance weights	ϑ_1_,ϑ_2_	0.1, 0.2
Coupling coefficient	𝓁	0.1
CACC gains	k_1_,k_2_,k_3_,k_4_	0.35, 0.90, 0.80, 1.00
Leader velocity feedback	k_L_	0.60
Desired spacing	d_0_	10 m
Evaluation window	T	5 s
Threshold level	α	0.005 (99.5th percentile of 100-run ensemble)
Integration step	N/A	0.02 s

**Table 2:** Observer design results.

Design	γ	η (Target)	Gain L (Row-Major)
*H*_∞_-only (Theorem 1)	0.0622	N/A	0.6592; 0.2668; 0.6389; 2.8960; 0.1612; 4.0304
Proposed ($\overset{‾}{\eta }=0.9$)	0.27	0.90	1.3698; −0.0732; 3.2769; −0.2907; 0.4879; 0.0671
Common settings	Gain bound ν = 5	P ≽ I	λ_k_ ∈ {0,0.38,1.38,2.62,3.62}

**Table 3:** Detection performance by attack class (proposed versus *H*_∞_-only baseline; detection rate over attacked samples, false-alarm rate measured on an independent attack-free ensemble, mean detection delay).

Attack Class	Method	DR (%)	FAR (%)	Delay (s)
Transient FDI	Proposed	100.0	0.46	0.00
	*H*_∞_-only	100.0	0.52	0.00
Zero-dynamics	Proposed	0.0	0.46	N/A
	*H*_∞_-only	79.2	0.52	0.10
DoS (link 2 → 3)	Proposed	48.8	0.46	5.12
	*H*_∞_-only	84.4	0.52	1.56
Replay	Proposed	100.0	0.46	0.00
	*H*_∞_-only	100.0	0.52	0.00
Transient FDI	Sliding mode [14,39]	100.0	99.84	0.02

The detection rate (DR) is the fraction of attacked samples inside the window with J_er,i_(t) above the threshold; FAR is measured on the independent attack-free validation ensemble (Section 4.5); delay is the mean time from attack onset to the first alarm. The higher zero-dynamics detection rate of the *H*_∞_-only baseline (79.2% versus 0.0%) reflects a threshold-scale effect: its quieter attack-free residual leads to a lower calibrated threshold, rather than a sensitivity advantage; see Section 4.4. The sliding-mode row reports the observer of [14,39] with its original literature parameters, without re-tuning.

**Table 4:** Zero-dynamics attack sweep (proposed observer): detection rate (%) against peak amplitude, decay rate, and injection vehicle; mean over three randomised realisations.

Peak Amplitude (m/s)	Decay Rate (1/s)	DR, Vehicle 1 (%)	DR, Vehicle 2 (%)	DR, Vehicle 3 (%)
0.03	0.5	0.0	0.0	0.0
0.03	1.5	0.0	0.0	0.0
0.03	3	0.0	0.0	0.0
0.1	0.5	52.4	52.5	54.1
0.1	1.5	13.7	31.0	15.9
0.1	3	0.0	0.0	0.0
0.3	0.5	77.1	72.7	78.7
0.3	1.5	56.5	57.1	57.1
0.3	3	52.1	52.9	52.7
1	0.5	98.2	97.7	98.4
1	1.5	63.6	65.9	66.1
1	3	58.0	61.4	61.1

**Table 5:** Method comparison and ablation across scenarios (DR in %, FAR in %, delay in s).

Configuration	DR (%)	FAR (%)	Delay (s)
Proposed (full)	100.0	0.46	0.00
*H*_∞_-only observer	100.0	0.52	0.00
Sliding-mode baseline [14,39]	100.0	99.84	0.02
Coupling removed (H = 0), transient	98.3	0.57	0.03
Coupling removed (H = 0), DoS	0.0	0.57	N/A
Coupling removed (H = 0), zero-dyn.	20.6	0.57	N/A
2× disturbance, nominal thresholds	N/A	16.43	N/A
N = 8 chain	100.0	0.47	0.00

**Table 6:** Packet-loss robustness of the proposed observer: false-alarm rate with the nominal and with recalibrated thresholds, and transient-attack detection rate, under independent Bernoulli loss on every V2V link.

Loss Probability *p*	FAR, Nominal Thresholds (%)	FAR, Recalibrated (%)	Transient DR (%)
0.01	0.50	0.49	100.0
0.05	0.50	0.49	100.0
0.10	0.50	0.49	100.0
0.20	0.50	0.49	100.0

**Table 7:** Static versus freeze-on-alarm adaptive thresholds under disturbance-level mismatch (proposed observer; κ_a_ calibrated to the nominal false-alarm rate). All rows share the same 30-run validation ensemble; the ×1.0 static value therefore differs slightly from the 100-run validated false-alarm rate of Section 4.3.

Disturbance Scale	FAR, Static Threshold (%)	FAR, Adaptive Threshold (%)
×1.0	0.53	0.53
×1.5	0.76	0.81
×2.0	16.43	2.09

## Data Availability

The original contributions presented in this study are included in the article. Further inquiries can be directed to the corresponding author.
